# Interaction of *Cis*- and *Trans*-RuCl _2_(DMSO)_4_ With Human Serum Albumin

**DOI:** 10.1155/MBD.2000.293

**Published:** 2000

**Authors:** Lilianna Trynda-Lemiesz, Henryk Kozlowski, Nikolas Katsaros

**Affiliations:** 1 Faculty of Chemistry University of Wroclaw F.Joliot Curie 14 Wroclaw 50-383 Poland; 2 NCRS ‘DEMOKRITOS’ Athens Greece

## Abstract

The interaction between cis- and trans- RuCl_2_(DMSO)_4_ and human serum albumin have been investigated through UV-Vis, circular dichroism, fluorescence spectroscopy and inductively couplet plasma atomic emission spectroscopy (ICP(AES)) method Albumin can specifically bind 1 mole of cis-isomer and 2 moles of the trans-isomer RuCl_2_(DMSO)_4_ complex. The interaction of RuCl_2_(DMSO)_4_ with HSA causes: a conformational change with the loss of helical stability of protein; the strong quenching of the Trp 214 fluorescence indicating that the conformational change of the hydrophobic binding pocked in subdomain IIA takes place; a local perturbation of the warfarin binding site and induce some conformational changes at neighbour domains, a changing of the binding abilities towards heme.


### INTERACTION OF *CIS*- AND *TRANS*-$RuCl_2(DMSO)_4$ WITH HUMAN SERUM ALBUMIN

Lilianna Trynda-Lemiesz*[1], Henryk Kozlowski[1], and Nikolas Katsaros[2]

[1]Faculty of Chemistry,University of Wroclaw, F.Joliot Curie 14,50-383 Wroclaw, Poland
[2]NCRS 'DEMOKRITOS', Athens, Greece

### Abstract

The interaction between cis- and trans- $RuCl_2(DMSO)_4$ and human serum albumin have been investigated through UV-Vis, circular dichroism, fluorescence spectroscopy and inductively couplet plasma atomic emission spectroscopy (ICP(AES)) method Albumin can specifically bind 1 mole of cis-isomer and 2 moles of the trans-isomer $RuCl_2(DMSO)_4$ complex. The interaction of $RuCl_2(DMSO)_4$ with HSA causes: a conformational change with the loss of helical stability of protein; the strong quenching of the Trp 214 fluorescence indicating that the conformational change of the hydrophobic binding pocked in subdomain IIA takes place; a local perturbation of the warfarin binding site and induce some conformational changes at neighbour domains, a changing of the binding abilities towards heme.

### Introduction

In recent years metal-based antitumor drugs have been playing a relevant role in antiblastic-chemotherapy, especially cis-DDP is regarded as one of the most effective anticancer drugs used in clinics. Among non platinum transition metal anticancer compounds, ruthenium complexes haveraised great interest. In a relatively early stage, the antitumor properties of ruthenium (II) with dimethyl sulfoxide (DMSO) as ligand, were reported, obviously selected for its neutrality and structural relation with cisplatin [1].

Compared to cisplatin the ruthenium(II) complex is less toxic and can be administrated in a remarcably higher maximum dose. Despite its octahedral geometry and the absence of amino ligands, cis-$RuCl_2(DMSO)_4$ presented some interesting analogies with cis-$PtCl_2(NH_3)_2$ (cisplatin), that is neutrality ,two cis-chloride ligands, high stability of 2+ oxidation state and high affinity for nitrogen donor ligands. In the later study, also the trans-isomer of this dmso complex proved to posses a comparable activity, and striking at much lower doses than the cis isomer [2]. Both isomers, cis-and trans-$RuCl_2(DMSO)_4$ were shown to posses antitumor and in particular, remarkable antimetastatic activity against some murine tumor models(P388 leukemia, platinum resistant P388, Lewis lung carcinoma, B16 melanoma [2-4].

Moreover the ruthenium complexes had an overall less pronounced host toxicity than cisplatin. Ruthenium(II) complexes interacts „in vitro" with DNA to form covalent bounds with the nucleobases, especially guanine N7[5].

Studies of the toxicity and antitumor activity of cis- and trans $RuCl_2(DMSO)_4$ show a significantly higher toxicity of trans isomer. In contrast, both complexes exhibit a significant antimetastatic activity, cis-$RuCl_2(DMSO)_4$ reduces the number and weight of spontaneus lung metastases by 46% and 52% respectively. The trans isomer is slightly more active than the cis one with an inhibition of 57% and 71% respectively. The antimetastatic activity of cis-DDP is only slightly more pronounced than that of trans-$RuCl_2(DMSO)_4$ [6].

The two complexes are very stable towards air oxidation in solution, and in both cases, in analogy to cisplatin, the dissociation eguilibrium is inhibited by extracellular chloride concentration. The chloride dissociation process that follows DMSO release is faster in the cis-isomer, due to the trans-effect of DMSO. Owing to the higher number of free coordination positions and, in particular, to the lower steric hindrance, the aquo-derivatives of trans-$RuCl_2(DMSO)_4$ are more reactive than those of the cis-isomer. In this way, the trans isomer is more capable of binding to biomolecules, and accordingly, much more cytotoxic than the cis-form. The study of the interaction of the complexes with imidazole confirmed that trans isomer turns out to be considerably more reactive then the cis isomer towards N-donor ligands [6].

To obtain an insight into the intravenously administered antitumor metal complexes, it is important to study the interaction of these with plasma proteins, especially albumin., which plays a central role in the molecular pharmacology of drugs used in the cancer chemotherapy.

Human serum albumin (HSA) interferes with certain anticancer agents, changing their biological activity and clinical effectiveness [7].

HSA is a single-chain 66 kDa protein, which is largely $\alpha$-helical, and consists of three structurally homologous domains, that assemble to form a heart- shaped molecule. Each domain is a product of two subdomains, which are predominantly helical and extensively cross-linked by several disulphide bridges [8]. Its amino-acid sequence contains a total of 17 disulphide bridges, one free thiol (Cys 34) and a single tryptophan (Trp 214). Albumin is known to bind and transport many ligands, including fatty acids, amino acids, steroids, metal ions, and a variety of pharmaceuticals [8,9]. The principal regions of ligand binding sites of albumin are located in hydrophobic cavities in subdomains IIA and IIIA, which exhibit similar chemistry. The binding locations have been determined crystallographically for several ligands [8]. The IIIA subdomain is the most active in accommodating of many ligands, as for example, digitoxin, ibuprofen and tryptophan. Aspirin show nearly equal distributions between binding sites located in IIA and III subdomains, while warfarin occupies a single site in IIA [10].

Ligand biding to one domain (II or III) induces distinct conformational changes in the other domain, as both subdomains share a common interface. Thus, the binding of particular drug molecule to serum albumin may change considerably binding abilities of HSA towards other molecules.

Previous studies [11,12] have demonstrated that the interactions of Ru(III) complexes with HSA causes distinct variations in protein conformation including a considerable decrease of the helical structure and a change of HSA binding abilities towards other molecules.

In this work we have performed extended studies on the interaction of human serum albumin with Ru(II) dimethylsulfoxide complexes

The effect between ruthenium complexes with albumin has been investigated through gel–filtration chromatography, UV/visible, CD, fluorescence spectroscopy and ICP(AES) method.

### MATERIALS AND METHODS

### Materials

The human serum albumin was obtained from Fluka Chem.Co. Warfarin and Sephadex-25 were purchased from Sigma. HSA concentration was determined by absorption spectrum, taking the absorbance of a 1mg/cm $^3$ at 280 nm as 0.55 [13].

Hemin chloride was used as obtain from Serva. Its concentration was evaluated spectrophotometrically in 0.01M NaOH, using absorption coefficient of 58.4mM$^{-1}$cm$^{-1}$ at 385nm [13].

Cis-and trans- $RuCl_2(DMSO)_4$ synthesized as described earlier [2] and was used in all experiments from a freshly prepared 2x10$^{-3}$ M aqueous solution.

In all the experiments a physiological buffer, pH 7.4 was used.

Complexes of albumin with ruthenium (II) compound were prepared by incubation of the reaction mixtures for 48 h at 37$^0$C; 48 h at37$^0$C and 5 days at 4$^0$C (7 days of incubation); 48 h at 37$^0$C and 12 days at 4$^0$C (14 days of incubation) in sterillized tubes.

### Methods

*Absorption and Difference Spectra* were recorded on SPECORD M-42 spectrophotometers, and CD spectra on a Jasco JK-600 spectropolarimeter. CD spectra were recorded over the range of 190-250 and 300-600 nm, using 0.1 and 1.0 cm cuvettes respectively. Secondary structure composition were calculated by Microsoft Dicroprot V2.4 program (by Gilbert Deleage, Institut de Biologie et Chemie des Proteines, Lyon, France).

Fluorescence measurements were carried out on an SLM AMINco SPF-500 spectrofluorimeter with the excitation and emission wavelength set at 298 and 350 nm (HSA) and 335 and 378 nm (warfarin).

The assays of ruthenium bounded per mol of HSA were performed with a SPECTROMETER 3410 (Inductively Couplet Plasma-Atomic Emission Spectroscopy, ICP-AES). HSA was allowed to react with ten fold molar excess of ruthenium complex for 14 days at pH 7.4.

The sample was than chromatographed on the Sephadex G-25 column (2x50 cm) equilibrated and eluted with physiological buffer pH 7.4.

The ruthenium content in selection fraction was determined by the ICP method.

### RESULTS AND DISCUSSION

### Binding of Ru(II) complexes to HSA

Fig.1 shows the binding of cis-and trans- $RuCl_2(DMSO)_4$ to HSA as a function of incubation time over a 14 days period. Binding of trans-isomer to HSA was the most effective one. When ruthenium(II) complexes were incubated at 10-fold excess to HSA, the adduct Ru/protein was formed for both cis – and trans-Ru(II), but the binding of trans isomer was the most effective case. Equilibrium was completed during 24h in the case of trans isomer (2 mole per mol of HSA) and 48h in the case of cis-$RuCl_2(DMSO)_4$ (1 mol per mol of HSA). Fig.1.

The essential difference between the two isomers is a kinetic one. In both cases, in analogy to cisplatin the dissociation equilibrium is inhibited by extracellular chloride concentration. Upon dissociation in water, trans-$RuCl_2(DMSO)_4$ releasees two DMSO molecules quicly (cis-isomer one DMSO molecule) and one Cl slowly. The higher number of free coordination positions and lower steric hindrance, causes,that the aquo-derivatives of trans-$RuCl_2(DMSO)_4$ are more reactive than those of the cis-isomer.

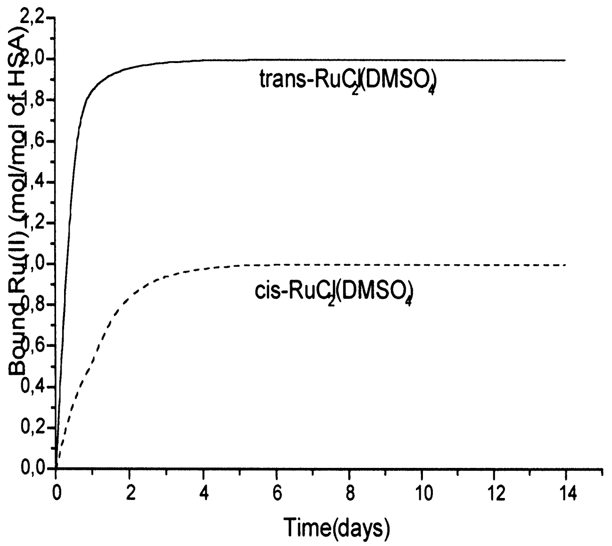

Figure1.The binding of ruthenium complexes to HSA as a function of incubation time, over a 14 day period. The ruthenium content of selected fractions were determined usingICP(AES) method.

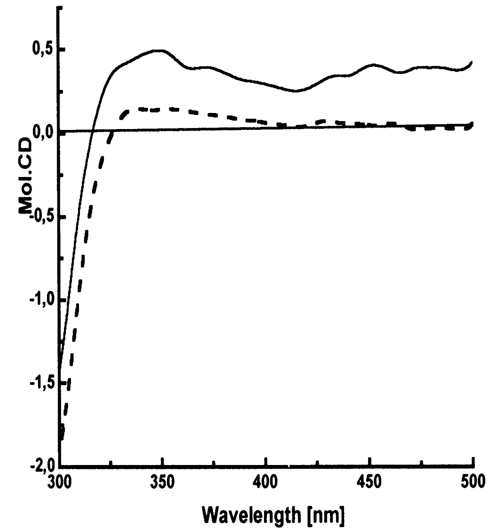

Figure 2. CD spectra in the visible region of cis-Ru(II)-HSA(- - -) and trans-Ru(II)-HSA(—) after 48h of incubation at $37^0$C. Mol.CD units: ($M^{-1}cm^{-1}$)

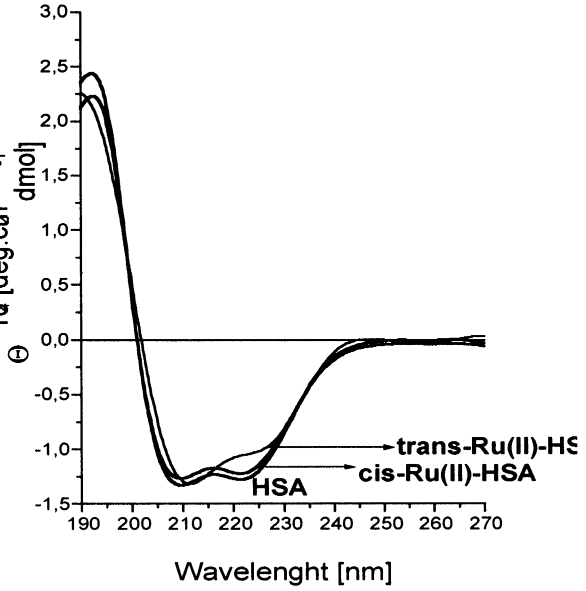

Figure 3A.CD spectra of HSA incubated for 48h with cis-and trans- $RuCl_2(DMSO)_4$ . Concentration of HSA: $8x10^{-6}$M.Molar ratio Ru/HSA, 2:1.

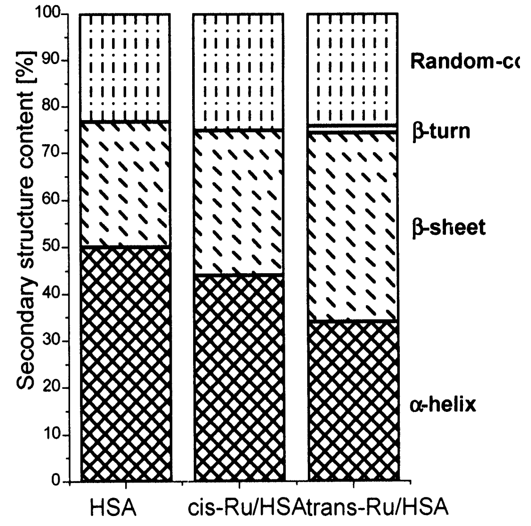

Figure 3B.Quantitative analysis of the secondary structure changes of HSA and Ru(II)-modified HSA, determined by DICROPROT V2.4 program.

This difference was confirmed earlier by studing their interaction with simple model molecules,imidazole [14].

Insights into the overall geometry can be found by examining the CD spectra of the $RuCl_2(DMSO)_4$ – HSA complexes. Circular dichroism spectroscopy is a technique particularly suited to prove specific binding of small chromophoric complexes to chiral macromolecules of high molecular weight.[15]. In the visible region (Fig 2) characteristic CD bands are seen at 330-350nm nm for both isomers and around 450 nm for trans-isomer, after 48h of the incubation at 37°C with HSA and separation by means of gel-filtration chromatography The spectra are characteristic of the formation of a specific adduct with the protein.

**Structural properties of the HSA modified with cis- and trans-$RuCl_2(DMSO)_4$**

In order to obtain information about the structural perturbation of HSA, the CD and fluorescence measurements are performed (Fig.3 and 4).

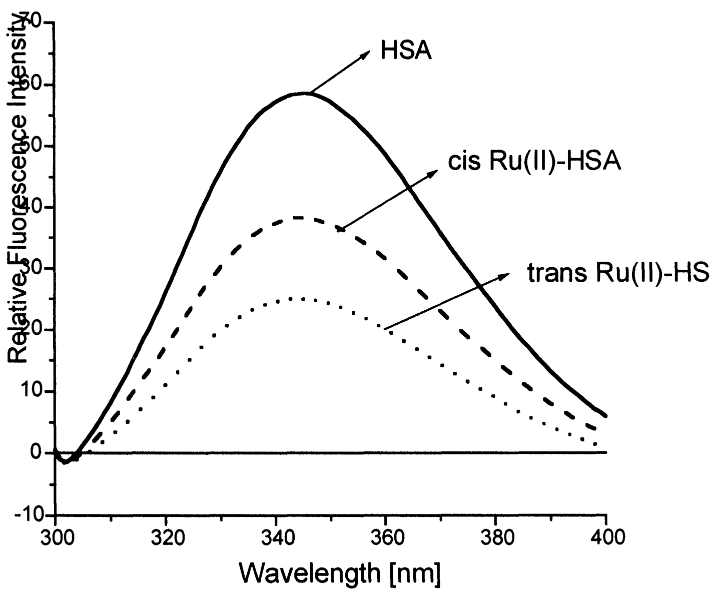

Figure 4.Fluorescence spectral changes of HSA incubated for 48h at 37 °C with cis- and trans-$RuCl_2(DMSO)_4$ at a molar ratio Ru/protein of 2. Concentration of HSA: $4x10^{-5}$M. Excitation at 298nm.

The albumin structure is predominantly α- helical, the number of helices in the structure is 28 [8]. CD spectra of HSA exhibit two negative bands in the ultraviolet region at 209 and 220 nm characteristic of an α-helical structure. (Fig.3A). The ruthenium(II) complexes binding results in protein secondary structural changes from that of the a- helix 50% (free HSA) to 43% (cis-isomer) and 33% (trans-isomer), β-sheet 26% (free HSA) to 32% (cis-isomer and 40% (trans-isomer) Fig.3B. The observed spectral changes indicate a partial unfolding of the protein structure and major alterations of the protein secondary structure from that of the α–helix to β-sheet conformation. Trans isomer comparison with cis seems to be sterically more favoured by a specific protein binding sites. From these results it is apparent that interaction of both isomers of ruthenium(II) with HSA causes a conformational change of the protein, with the loss of helical stability, as found for the platinum[16], ruthenium(III) [11,12] and rhodium complexes [17].

Tryptophan fluorescence is most frequently examined among the three intrinsic aromatic fluorescing groups in HSA molecules to obtain information about conformational changes [18].

Figure 4 shows typical changes of fluorescence intensity of the reaction mixture in which cis – and trans-$RuCl_2(DMSO)_4$ complexes and HSA were incubated for 48h at 37°C. When HSA is excited at 300-298 nm, a fluorescence efficiency around 350 nm reflects changes in the microenvironment of tryptophan residue. The relative fluorescence intencity of ruthenium-bound HSA decreased to about 55% (cis-Ru) and to about 36% (trans- Ru), when compared to the native state, suggesting that the binding occurs at the tryptophan residue or its proximity. No change of the fluorescence intensity was observed for the control HSA solution over an incubation period. It is very probable that the bindings of cis-and trans- $RuCl_2(DMSO)_4$ result in tryptophan exposure to a polar environment. Much more effective quenching is observed in the case of trans-isomer , when the amount of ruthenium bound to protein reaches twice.

The strong quenching of the Trp214 fluorescence clearly indicate that the conformation of the hydrophobic binding pocket in subdomain IIA is significantly affected by the ruthenium binding. It may be due to cis-and trans- $RuCl_2(DMSO)_4$ binding to His 242 or His 246 being close to Trp 214 in the domain IIA binding pocket, and possibly the other His residues located near this region.

**Influence of ruthenium (II) on the warfarin and hemin binding to HSA**

To provide further information about ruthenium binding site, the binding of warfarin, a site I marker, was also investigated.

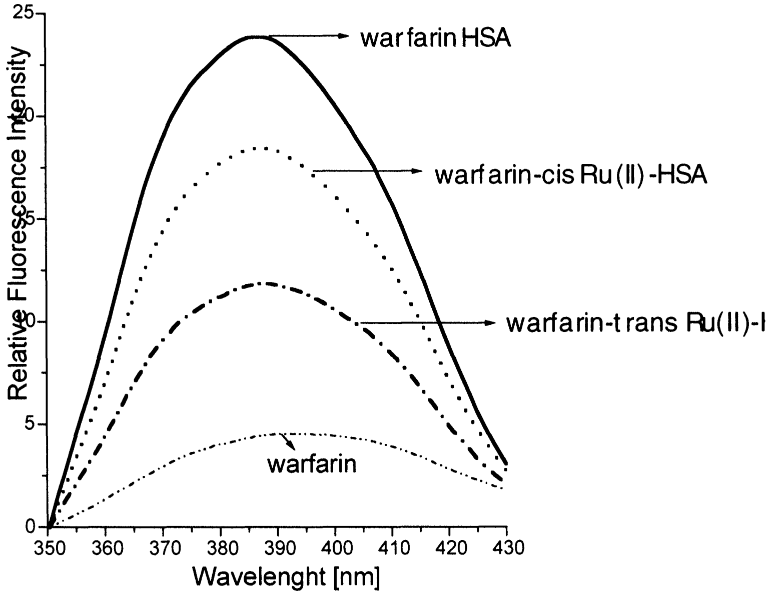

Figure 5. Relative fluorescence changes of warfarin with ruthenium – treated HSA. Excitation at 335nm and emission at 378 nm. Warfarin was incubated for 1h at $37^0C$ with HSA(—), cis-Ru(II)-HSA(-----) and trans Ru(II)-HSA(-_-_-_) at molar ratio 1:2 Concentrations of HSA:$4x10^{-5}$M; warfarin:$8x10^{-5}$M.

The warfarin is one of the best characterised drug as far as the binding sites are concerned [19]. Its site I is located in subdomains IIA near Trp-214 [10].

Warfarin has a weak fluorescence at 378 nm when excited at 335 nm, and the addition of HSA induced an increase in fluorescence intensity when warfarin binds to a single site in the protein [20].

Fig.5 shows the relative fluorescence changes of warfarin with $RuCl_2(DMSO)_4$ treated HSA.

Cis- and trans- $RuCl_2(DMSO)_4$ complexes were obtained after 48h incubation at $37^0C$.

The strong fluorescence decrease of warfarin bound to protein indicates the reduction in the warfarin binding capacity at the primary binding site of HSA. It is very likely that ruthenium binding occurs at the Trp-214 residue or in its proximity within a warfarin site I, located in subdomains IIA. Warfarin and many other drugs (aspirin$^R$, 5-iodo-salicylic acid, triiodobenzoic acid) were found to bind preferentially in IIA domain.

The heme-albumin and bilirubin-albumin complexes appears as an intermediate in the plasma heme and bilirubin degradation processes. Earlier studies on heme-HSA interactions have shown that the protein posses only one strong binding site for heme molecules although some weaker sites have been also proposed [21,22]. The comparative study of the heme interaction with whole HSA as well as its three recombinant domains [23] has indicated that primarys binding site is located in HSA-domain I and additinal secondary binding sites is placsd in in the HSA-domain II. The results shown in Fig.6 clearly indicate that cis-and trans- $RuCl_2(DMSO)_4$ complexes bound to HSA modifies only insignificant the primary heme binding site.

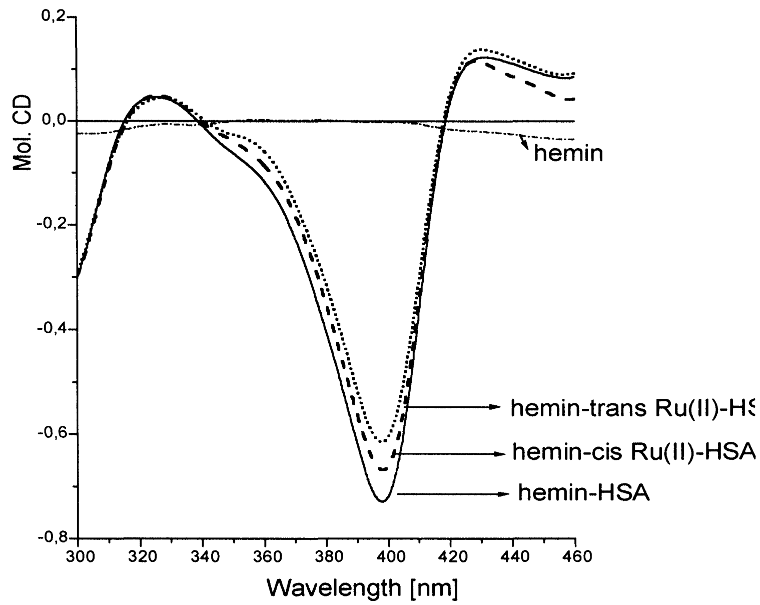

Figure 7. Effect of cis- and trans $RuCl_2(DMSO)_4$ -HSA on the visible CD spectrum of HAS hemin complex: hemin-HSA(_); hemin-cis Ru(II)-HSA(- - -); MolCD units: $[M^{-1}cm^{-1}]$.

When only the strongest binding sites are occupied, the characteristic CD signal of the heme with native albumin appears. The method is based on the observation that an optical activity arises from dissymmetry in the ligand induced by its binding to the protein, since the free ligand has either no asymmetric center.Hemin is an optically inactive molecule, however, the strong interactions with protein induced chirality and its intramolecular transition could be observed in the CD spectra.

The changes in the protein conformation within the hemin binding region indicates that cis-and trans-$RuCl_2(DMSO)_4$ binding can induce some conformational changes at neighbour domains.

### Conclusions

The interaction of anticancer drugs with blood constituents, particularly with serum albumin may have a major influence on drug pharmacology and efficacy. [7]

As it was already shown [24,25] about 80% of platinum and ruthenium complexes in the blood plasma is bound to serum proteins (primary albumin). Thus, it is important to understand the effect of this binding on the structure of the protein and interactions with other biologically relevant ligands (e.g. drugs).

The results presented above clearly indicate that albumin can specifically bind 1 mole of cis-isomer and 2 moles of the trans-isomer $RuCl_2(DMSO)_4$ complex according to ICP(AES) method.. The interaction of $RuCl_2(DMSO)_4$ with HSA causes: a conformational change with the loss of helical stability of protein; the strong quenching of the Trp 214 fluorescence indicating that the conformational change of the hydrophobic binding pocked in subdomain IIA takes place; a local perturbation of the warfarin binding site. and induce some conformational changes at neighbour domains, a changing of the binding abilities towards heme.

**Acknowledgements.** This work was supported by the Polish State Committee for Scientific Research (KBN 4P05F01615). This work was performed within COST D8/0018/98 project.

**References**

1.T.Giraldi , G.Sava ,G.Bertoli, G.Mestroni, G.Zassinovich. *Cancer Res.* **37**, .(1977) 2662.
2.E.Alessio, G.Mestroni, G.Nardin, W.M.Attia, M.Calligaris, G.Sava, S.Zorzet. *Inorg.Chem.* **27** (1988) 4099.
3. J.S. Jaswal, S.J. Rettig,B.R. James, *CanJ.Chem.* **68**(1990) 1808.
4.G.Sava, S.Pacor,F.Bregant,V.Ceshia, E.Luxich, E.Alessio, G.Mestroni, *Pharmacology* (*Life Sci.Adv.*) **9**(1990) 79.
5.G.Esposito, S.Cauci, F.Fogolari, E.Alessio, M.Scocchi, F.Quadrifoglio, P.Viglino, *Biochemistry* **31** (1992) 7094.
6.G.Mestroni,E,Alessio,G.Sava, S.Pacor, M.Coluccia, Progress in Clinical Biochemistry and Medicine, vol.10, Springer-Verlag Berlin Heidelberg 1989, 71.
7. T. Takahashi, T.Ohnuma, S.Kavy, S.Bhardwaj, J.F. Holland, *Br.J.Cancer* **41** (1980) 602.
8. D.C.Carter, J.X. Ho, *Adv.Protein Chem.* **45** (1994) 153.
9. T. Peters, Jr.,*Adv.Protein Chem.* **37**, (1985) 161.
10. X.M. He, D.C. Carter, *Nature* **358** (1992) 209.
11. L.Trynda-Lemiesz, B.K.Keppler, H.Kozlowski, *J.Inorg.Biochem.* **73** (1999) 123.
12. L.Trynda-Lemiesz, A.Karaczyn, B.K.Keppler, H.Kozlowski, *J.Inorg.Biochem.* **78** (2000) 341.
13. G.H.Beaven, S.H.Chen, A d'Albis, W.B. Gratzer, *Eur.J.Biochem.* **41** (1974) 539.
14. M.Henn, E.Alessio, G.Mestroni, M.Calligaris, W.M.Attia, *Inorg.Chim.Acta* **187** (1991) 187.
15. S. F. Mason, "Molecular Optical Activity and the Chiral Discrimination" Cambridge University Press, Cambridge, UK (1982).
16. J.F..Neault, H.A.Tajmir-Riahi, *Biochim.Biophys.Acta* **1384** (1998) 153..
17. L.Trynda-Lemiesz, F.Pruchnik, *J.Inorg.Biochem.* **66** (1997)187.
18. D. Freifelder, Physical Biochemistry: Applications to Biochemistry and Molecular Biology, W.H. Freeman, San Francisco, 1976, pp.410-443.
19. O.J.M. Bos, J.P.M.Remijn, J.E.Fisher, J.Witing, L.H.M. Janssen, *Biochem Pharmac.* **37** (1988) 3905.
20. K.J.Fehske, W.E.Muller, U.Wollert, Mol.Pharmacol.16 (1979) 778.
21. T. Peters, Jr., *Clin.Chem.* **23** (1977) 5.
22. Z.Hrkal, M.Kodiczek, Z. Vodrazka, B. Meloun, and L.Moravek,. *Int.J.Biochem.* **9** (1978) 349.
23. M.Dockal, D.C.Carter, F.Rüker, *J.Biol.Chem.* **274** (1999) 29303.
24. F.Kranz, N.Mulinacci, L.Messori, I.Bertini, B.K.Keppler, Metal Ions in Biology and Medicine, J.Anastassopoulou, Ph.Collery, J.C.Etienne, and Th.Theophanides, eds., J.Libbey Eurotext, Paris, 1992 vol.2, pp. 69-74
25. W.C. Cole, W.Wolf, *Chem-Biol.Interactions* **30** (1980) 223.

**Received: June 28, 2000 - Accepted: August 3, 2000 -**
**Received in revised camera-ready format: December 18, 2000**

